# Antimicrobial Susceptibility Patterns and Genetic Diversity of *Campylobacter* spp. Isolates from Patients with Diarrhea in South Korea

**DOI:** 10.3390/microorganisms12010094

**Published:** 2024-01-02

**Authors:** So Yeon Kim, Dongheui An, Hyemi Jeong, Jonghyun Kim

**Affiliations:** 1 Division of Zoonotic and Vector-Borne Disease Research, Center for Infectious Diseases Research, Korea National Institute of Health, Cheongju 28160, Republic of Korea; sykim0217@korea.kr; 2 Division of Clinical Microbiology, Department of Laboratory Medicine, Seegene Medical Foundation, Seoul 04805, Republic of Korea; amorvie74@mf.seegene.com (D.A.); jhm721@mf.seegene.com (H.J.)

**Keywords:** *Campylobacter jejuni*, *Campylobacter coli*, antimicrobial resistance, multilocus sequence typing, diarrhea patients

## Abstract

This study aimed to characterize the latest antimicrobial resistance patterns and genetic diversity of *Campylobacter* spp. isolated from patients with acute diarrhea in Korea. In total, 371 clinical isolates (361 *Campylobacter jejuni* and 10 *Campylobacter coli*) were collected from patients with diarrhea in 106 medical institutions of six provinces during the seasonal peak (April–September 2022) in South Korea. We then assessed their antimicrobial susceptibility to eight antimicrobial agents and performed multilocus sequence typing (MLST). This study investigated the antimicrobial resistance (AMR) profiles to tetracycline (32.3%), nalidixic acid (64.9%), and ciprofloxacin (83.3%), confirming high levels of the latter even after its Korean ban in 2010. However, tetracycline resistance displayed a decreasing trend. Alternatively, significantly lower resistance rates to clindamycin (0.8%), azithromycin (0.53%), erythromycin (0.53%), and gentamicin (0.53%) as well as absolute susceptibility to florfenicol (0%) were observed. Four *C. jejuni* and three *C. coli* isolates (7/371, 1.88%) were classified as multidrug-resistant (MDR) to at least three antimicrobial classes. MLST identified a high genetic diversity with 21 clonal complexes (CCs) and sixty-six sequence types (STs), including eight novel STs. The high CC frequency of *C. jejuni* comprised CC21 (37.7%), CC22 (13.8%), and CC206 (9.4%), while *C. coli* was predominated by CC828 (90%). The high CC21 and CC828 strain prevalence in this study was consistent with their worldwide distribution. This study highlights that quinolone- and tetracycline-resistant *Campylobacter* circulate in Korea with diverse genotypes, providing important information that could contribute to controlling and preventing increasing antimicrobial resistance in patients.

## 1. Introduction

*Campylobacter* species are a zoonotic pathogen causing foodborne diarrheal diseases worldwide, with *Campylobacter jejuni* and *Campylobacter coli* being the most important pathogenic species, responsible for approximately 90% of human infections [1,2]. In 2021, the European Center for Disease Prevention and Control reported approximately 129,960 human campylobacteriosis cases [3]. The main *Campylobacter* transmission route to humans is contaminated food handling, preparation, and consumption, especially that of poultry origin [4]. Although contamination is the primary cause of retail raw poultry, it can be transmitted to humans through cross-contamination on cutting boards, knives, cucumbers, and hands while handling contaminated poultry at home or in restaurants [5].

*Campylobacter* identification from patient stool is difficult because of the specific media and culture conditions. Isolation and cultivation of *Campylobacter* requires difficult culture conditions because of microaerophilic conditions and cultivation in a specific medium, and maintenance culture of the pathogen is difficult compared with other Gram-negative bacteria (pathogenic *E. coli*). In the case of *Campylobacter jejuni* NCTC11168, the doubling time in rich media (blood-based agar or MH agar) is 112 min under microaerobic conditions, which is slower than in *E. coli* (~20 min, aerobic condition). The most problematic aspect is that the positive culture rate of *Campylobacter* spp. decreases in inverse proportion to the time the sample is exposed to oxygen. Placing specimens in the transport medium is thought to prolong organism survival, but the length of successful storage is poorly defined [6].

Thus, fewer studies are available on clinical isolates than on poultry and food isolates from Korea. Although *Campylobacter* is reportedly an important food poisoning causative agent along with *Escherichia coli* and *Salmonella*, its importance has been evaluated less due to its low isolation rate in Korea [7,8]. Most campylobacteriosis cases are usually self-limiting, without any requirement for hospitalization and antimicrobial treatment. However, therapy is required in children with fever and increasing bloody diarrhea, as well as in elderly or immunocompromised patients with severe and prolonged systemic disorders [9,10]. In severe cases, macrolides (erythromycin) and fluoroquinolones (ciprofloxacin) are considered first- and second-line antimicrobials, respectively, for human *Campylobacter* infection treatment [11].

Antimicrobial overuse both in animals and in humans has led to increased antibiotic-resistant *Campylobacter* populations [12]. Therefore, *C. jejuni* resistance monitoring is highly relevant to public health. In particular, the widespread and indiscriminate antibiotic supplementation of poultry feed increases resistant bacterial emergence, especially that of multidrug-resistant bacteria, causing serious problems in human treatment and prevention [13]. In the European Union, antibiotic use was banned, except for disease treatment, in 2006 [14], starting with Sweden even earlier in 1986 [15]. In Korea, antibiotic supplementation of formulated feed was banned in July 2010 [16]. Since changes in antibiotic resistance could appear in various ways depending on the antibiotic type or investigation period and region, assessing antibiotic resistance distribution patterns is pivotal for efficient antibiotic use [16]. Therefore, developed countries (e.g., Denmark, Japan, and Canada) are continuously investigating antibiotic resistance against pathogenic bacteria at the national level [17,18,19].

To date, multiple studies have addressed *Campylobacter* antibiotic resistance distribution from domestic poultry-derived isolates [20,21]. However, studies on clinical antibiotic resistance distribution and *Campylobacter* clonal characteristics in patients with acute diarrhea after the ban of antibiotic supplementation of formulated feed remain scarce.

Thus, this study investigated the antibiotic resistance distribution in clinical patients and performed *Campylobacter* spp. epidemic clonal analysis in patients with acute diarrhea, systematically collecting samples from more than 100 medical institutions in six regions nationwide in South Korea.

## 2. Materials and Methods

### 2.1. Bacterial Isolate Collection

In total, 352 and 19 *Campylobacter* spp. isolates from fecal samples and rectal swabs, respectively, were collected from patients with diarrhea and campylobacteriosis from 106 medical institutions in six provinces (Seoul, Gyeonggi, Chungcheong [Chungbuk and Chungnam], Gyeongsang [Gyeongbuk and Gyeongnam], Jeolla [Jeonbuk and Jeonnam], and Gangwon/Jeju) in South Korea between April and September 2022. Of the 33,511 PCR-tested cases for the causative agent of acute diarrhea, 2492 *Campylobacter* spp.-positive specimens (positive rate: 7.4%), 361 and 10 strains being identified as *C. jejuni* and *C. coli*, respectively, were confirmed (371 isolates/2492 positive cases, isolation rate 14.9%). In order to collect unbiased strains nationwide, the minimum collected strain number of 15 or more from each of the six regions was set (Seoul, Gyeonggi, Jeolla, Chungcheong, Gyeongsang, Gangwon, and Jeju) (Table 1). Thus, strains nationwide based on isolation from single patients were collected.

### 2.2. Identification and Growth Conditions

The strains were cultured on blood-based agar with 5% defibrinated sheep blood and incubated at 42 °C for 48 h under microaerobic conditions (85% nitrogen, 10% carbon dioxide, and 5% oxygen) [22]. Then, the colonies were subcultured on the antibiotic supplement (cefoperazone 16 mg and amphotericin 5 mg; Oxoid, Basingstoke, UK)-containing modified charcoal cefoperazone desoxycholate agar (mCCDA, Oxoid) and incubated at 42 °C for 48 h under microaerophilic conditions [23]. We stored the *Campylobacter* isolates as frozen stocks at −80 °C in Tryptic Soy Broth containing 20% glycerol supplemented with 5% defibrinated sheep blood. All presumptive *Campylobacter* spp. isolates at the species level were identified (i.e., *C. jejuni* and *C. coli*) using the Matrix-Assisted Laser Desorption/Ionization biotyper (Bruker) and VITEK 2 (Biomerieux) [24]. The genomic DNA were extracted using the MagNA Pure 96 System (Roche Diagnostics, Pleasanton, CA, USA), fully automated, for high throughput nucleic acid purification according to the manufacturer’s instructions. The eluted DNA was stored at −20 °C for further MLST screening.

### 2.3. Antimicrobial Resistance Testing

Phenotypic screening was performed with minimum inhibitory concentrations (MICs) to macrolides (azithromycin [AZY] and erythromycin [ERY]), fluoroquinolones (ciprofloxacin [CIP]), quinolone (nalidixic acid [NAL]), lincosamides (clindamycin [CLI]), phenicol (florfenicol [FFN]), tetracyclines (tetracycline [TET]), and aminoglycosides (gentamicin [GEN]) as previously described using a commercial microdilution tool (Sensititre™ plates; Sensititre™ *Campylobacter* plate–CAMP2, Trek Diagnostic Systems, East Grinstead, UK) and following the manufacturer’s instructions. All *Campylobacter* isolates were grown for 24 h at 42 °C under microaerobic conditions (10% CO_2_, 5% O_2_, and 85% N_2_), generated using the MGC GasPak Sachets system (MGC, Japan). The susceptibility results were interpreted using the epidemiological cutoff values set by the U.S. National Antimicrobial Resistance Monitoring System (NARMS) for *Campylobacter* isolates (https://www.cdc.gov/narms/antibiotics-tested.html, accessed on 31 October 2023) [24]. The MIC breakpoints were interpreted for resistance according to the NARMS-2019 recommendations for *C. jejuni* and *C. coli* as follows: AZY ≥ 0.5 mg/L (*C. jejuni*) and 1 mg/L (*C. coli*); ERY ≥ 8 mg/L (*C. jejuni*) and 16 mg/L (*C. coli*); CIP ≥ 1 mg/mL, NAL ≥ 32 mg/mL, and TET ≥ 2 mg/L (*C. jejuni*) and 4 mg/L (*C. coli*); GEN ≥ 4 mg/mL. The *C*. *jejuni* strain ATCC 33560 was used as a quality control. Multidrug resistance (MDR) was defined as resistance to at least three unrelated antimicrobial classes [11].

### 2.4. Clonal Population

All *C. jejuni* and *C. coli* isolates were characterized by multilocus sequence typing (MLST) based on primers for seven housekeeping genes of each isolate, including *aspA*, *glnA*, *gltA*, *glyA*, *pgm*, *tkt*, and *uncA* [25]. Allele numbers for each housekeeping gene, sequence types (STs), and clonal complexes (CCs) were assigned by submitting the DNA sequences to the *Campylobacter* MLST database (https://pubmlst.org/campylobacter/, accessed on 31 October 2023) at the University of Oxford. The PubMLST algorithm was used to assign CCs that were conditional on having at least five or more identical alleles regarding the founder allelic profile. Therefore, Phyloviz 2.0 software was used to analyze the genetic relationships between isolates constructed from MLST profiles [26].

### 2.5. Statistical Analysis

Statistical analysis was performed using GraphPad Software (version 8.3.0; GraphPad Software, San Diego, CA, USA). Fisher’s exact test was used to compare differences in the ratios of infection between patient gender and in the ratios of resistance between the two *Campylobacter* species.

## 3. Results

### 3.1. Campylobacter spp. Isolation and Identification from Patients with Diarrhea

**Figure 1:**
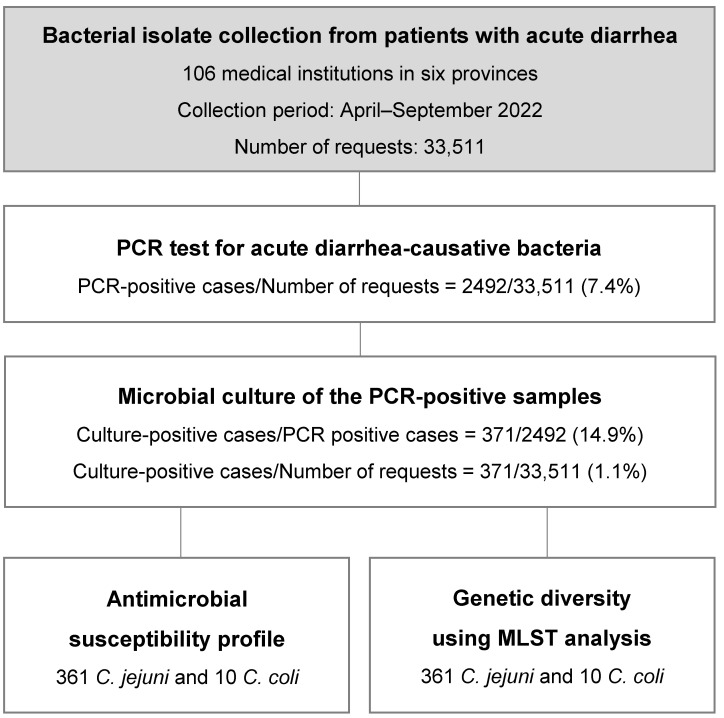
Study workflow diagram.

In Korea, the isolation rate of *Campylobacter* spp. was high from early summer to early fall, i.e., from May to September [27]. Because the average isolation rate reached its highest average level in July and August, sample collections were performed for the entire isolation period of 6 months between April and September 2022 in Korea. The samples were transported at −20 °C to the laboratory and processed within 24 h. Of the 33,511 total PCR requests for causative agents of acute diarrhea in human patients, 2492 *Campylobacter* PCR-positive cases (7.4%, 2492/33,511) and isolated 371 *Campylobacter* spp. strains (14.9%, 371/2492 PCR-positive cases) (Figure 1) were diagnosed. The isolation rates by region were as follows: 35/132, 88/555, 39/254, 75/691, 104/749, and 30/111 positive culture/PCR-positive cases for Seoul (26.5%), Gyeonggi (15.9%), Chungcheong (10.9%), Jeolla (15.0%), Gyeongsang (13.9%), and Gangwon and Jeju (27.0%), respectively (Table 1).

### 3.2. Campylobacter spp. Antimicrobial Resistance

Azithromycin and ciprofloxacin are recommended as the first- and second-choice antibiotics for *Campylobacter* treatment [2,18]. All 371 *Campylobacter* isolates were screened for antimicrobial susceptibility to eight antimicrobials, yielding high-level resistance to ciprofloxacin, nalidixic acid, and tetracycline, with antimicrobial resistance rates as follows in *C. jejuni* and *C. coli*, respectively: ciprofloxacin 82.6% (n = 298), nalidixic acid 64.1% (n = 231), and tetracycline 27.7% (n = 113); ciprofloxacin 100% (n = 10), nalidixic acid 90% (n = 9), and tetracycline 70% (n = 7) (Table 2). In contrast, both *C. jejuni* and *C. coli* exhibited significantly lower resistance rates to macrolides (2/371, 0.53%), lincosamides (3/371, 0.8%), and aminoglycosides (2/371, 0.53%). Moreover, no florfenicol-resistant *C. jejuni* and *C. coli* or gentamicin-resistant *C. jejuni* were identified in this study (Table 2).

The most prevalent resistance was observed upon the combination of ciprofloxacin and tetracycline (118/371, 31.8%). Seven of all the isolates were classified (7/371, 1.88%) as multidrug-resistant (MDR) (i.e., four and three *C. jejuni* and *C. coli* isolates, respectively) to three or more antimicrobial classes (Table 3). *Campylobacter* spp. isolates from patients with diarrhea developed their main antimicrobial resistance against the combination of fluoroquinolones and tetracyclines.

### 3.3. Campylobacter spp. Genetic Diversity

The *Campylobacter* spp. strains sequenced in this study were grouped by species (Table 4). The 361 *C. jejuni* isolates exhibited a significant variety of 20 CCs and 57 STs (including eight new). These eight STs (ST2274, ST4240, ST5046, ST5229, ST12268, ST12597, ST12636, and ST12656) from ten isolates did not belong to any known CCs (unassigned CC). The MLST analysis showed that *C. jejuni* strains of three major CCs (CC21, CC22, and CC206) were dominant in the patients with diarrhea examined in this study. The CC21, including 12 STs (ST19, ST21, ST50, ST298, ST451, ST760, ST806, ST982, ST3128, ST3293, ST4253, and ST6500), was identified in 37.7% (136/361) of the human *C. jejuni* isolates, followed by CC22 including one ST (ST22), in 13.8% (50/361); finally, we identified CC206, including four STs (ST122, ST227, ST572 and ST3335), in 9.4% (34/361). Therefore, the most commonly isolated CC was CC21 (136/361, 37.7%) in the 361 human *C. jejuni* isolates from South Korea. Eight new STs were identified as novel allele-type combinations based on the PubMLST database, which did not belong to any known CCs.

The *C. coli* (n = 9) strains were represented by eight STs (ST830, ST854, ST860, ST872, ST9201, ST9867, ST12600, and ST12613) and could be grouped within the same CC828. One of them (ST10873) did not belong to any known CCs (unassigned CC). Additionally, two novel strains were identified that have not been previously described in the MLST database (Table 4).

**Figure 2:**
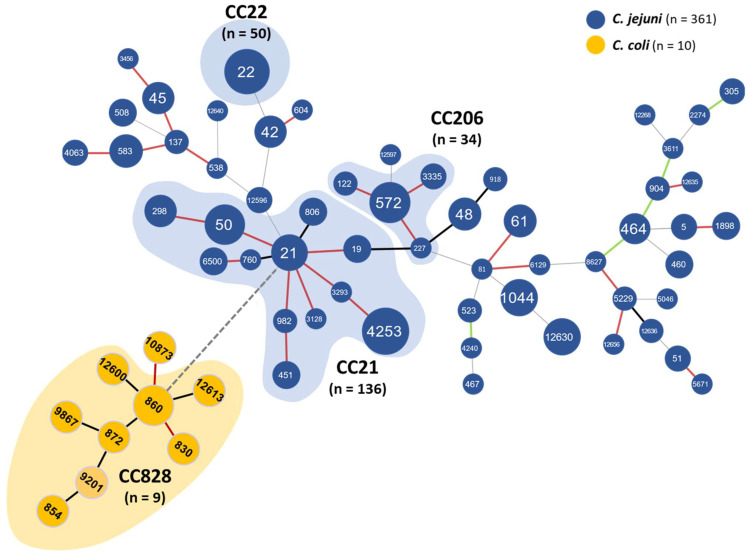
Minimum spanning tree constructed with the conventional 7-locus MLST profiles of 371 *Campylobacter* isolates. Each circle size is proportional to the number of isolates, with the sequence type (ST) labeled. Differences of 1, 2, 3, and >3 loci between STs are represented by solid red, black, green line, and gray lines, respectively. A clonal complex (CC) is designated for STs among which an ST shares five loci or more with the closest one.

In this study, our MLST identified eight and two STs for *C. jejuni* and *C. coli*, respectively. Our MLST data-based minimum spanning trees for the *C. jejuni* and *C. coli* isolates cluster analysis indicates that our human campylobacteriosis-related *Campylobacter* isolates belong to diverse clones (Figure 2).

## 4. Discussion

This clinical-focused study aimed to compensate for the lack of campylobacteriosis-related research in domestic clinical patients. Therefore, isolates from patients with acute diarrhea treated at 106 medical institutions in six regions nationwide in South Korea were systematically collected and identified, and their antimicrobial resistance patterns and epidemic clones were investigated. Laboratory monitoring of acute diarrhea in Korea confirmed that *Campylobacter* is the third most common pathogen, following pathogenic *Escherichia coli* and *Salmonella* [27]. However, *Campylobacter* pathogenicity is evaluated as less important because of its difficult isolation and culture due to complex culture conditions and the low isolation rate [28].

This study included 37,821 medical institutions nationwide, of which 4845 were transacted by the Seegene Medical Foundation (Seoul, Republic of Korea), accounting for 12.8% of the nation. Based on the highest *Campylobacter* isolation rates in Korea during the summer months (i.e., June–September) [27], fecal samples from patients with diarrhea were collected between April and September 2022 and pathogen isolation and identification was performed. The susceptibilities of the collected 361 *Campylobacter* isolates to eight antibiotics were tested, yielding the highest-to-lowest resistance to ciprofloxacin (83.3%), nalidixic acid (64.9%), tetracycline (32.3%), clindamycin (0.8%), azithromycin (0.53%), erythromycin (0.53%), gentamicin (0.53%), and florfenicol (0%).

The PCR-positive rate was 7.4% among the total requests (2492/33,511), the pathogen isolation rate from the PCR-positive patients was 14.9% (371/2492), and the pathogen isolation rate from the total requests was 1.1% (371/33,511) (Figure 1). This result was similar to other domestic data of Ryoo [8], demonstrating that the proportion of *Campylobacter* spp. among domestic acute gastroenteritis cases increased steadily between 2007 and 2019, accounting for 0.3–1.5%. *Campylobacter* spp. is reportedly the most common causative agent among patients with gastroenteritis [8]. However, different from European or North American patterns, it is the third most common causative agent in Korea, the reasons for which are unclear. Infections occurred most frequently in children (of 1–9 years) to young adults (of 15–29 years) (Supplementary Figure S1), indicating a pattern similar to the results of studies in Denmark and others [29,30].

In this study, the Gangwon/Jeju region had the highest PCR positivity rate (10.2%) and *Campylobacter* spp. isolation rate (27%), and the Seoul region had the lowest PCR positivity rate (4.0%) but a high isolation rate (26.5%). It is not believed that poultry consumption is particularly high in the Gangwon/Jeju region, nor that sanitary conditions are particularly poor. However, in the Gangwon/Jeju region, the hospitals where samples were collected account for a larger proportion of samples collected through tertiary hospitals than the other regions, resulting in more severe symptoms than patients who visited primary and secondary hospitals. This is because PCR and pathogen isolation were performed on samples that were present. In the case of Seoul, it is located in the same region as the laboratory and was conducted within 12 h from sample collection to the start of the experiment; therefore, the samples were exposed to oxygen for a shorter time than in other regions. Therefore, the isolation rate of pathogens is high. According to a report by Cho et al., the incidence rate of *Campylobacter* spp. was reported to be 1.23 times higher in rural regions than in urban regions [31]. By contrast, rates of 2.46% of *Campylobacter* spp. isolated in city regions and 0.58% in rural regions have been reported [27]. However, herein, rather than these regional characteristics, differences such as the severity of patient symptoms or delivery time to the laboratory after specimen collection are considered as the most important factors.

Campylobacteriosis is usually self-limiting and rarely requires antibiotic treatment, except for severe or prolonged cases [1,2]. Fluoroquinolones are broad-spectrum antimicrobials for treating a multitude of infections, including undiagnosed diarrhea cases, as priority therapy in Korea [32]. Therefore, increased ciprofloxacin resistance is considered a major public health concern. Such high antibiotic resistance might be closely related to antibiotic consumption on commercial farms. It was banned in 2010 because of the indiscriminate misuse of antibiotics, problems with antibiotic residues, and an increase in antibiotic multidrug resistant strains, and the use of commercial laying hens was banned in July 2017 [33]. However, the fact that consumption is not decreasing is because of customary use in farms and anxiety about a decrease in income due to non-use. The annual antibiotic consumption in Korea is 3 and 13.6 times higher than that in the United States and the European Union, respectively [13].

In Korea, antibiotic use in commercial animals was banned in 2010 in an effort to suppress the increase in fluoroquinolone resistance [23]. Nevertheless, from 2009 to 2013, the nalidixic acid resistance rate was 100% for human isolates and 95.2% for animal isolates, and the ciprofloxacin resistance rate was 96.8% for human isolates and 92.9% for animal isolates [34]. The fluoroquinolone resistance of *C. jejuni* was ciprofloxacin (89.4 to 100%) and nalidixic acid (90.9 to 100%), and there was no decreasing trend of antibiotic resistance [35,36]. Even our study result could not confirm a significant antibiotic resistance reduction against ciprofloxacin (83.3%) and nalidixic acid (64.9%), and maintenance of a high-level antibiotic resistance was described (Table 2). *C. jejuni* isolates from poultry meat yielded similar results. In the case of ciprofloxacin, chicken and duck meat-derived samples displayed 83.3–85.6% and 85.6–87.8% resistance, respectively. For nalidixic acid, chicken and duck meat-derived samples exhibited 92.9–97.8% and 92.7–97.8% resistance, respectively. So, the impact of banning antibiotics appears to be insignificant [21,37].

These results could be obtained potentially because mass fluoroquinolone use is still allowed for poultry in Korea. In particular, enrofloxacin displays the highest proportion as a poultry treatment [38]. Enrofloxacin is well absorbed when orally administered to poultry, and compared to first-generation nalidixic acid, it has excellent bioavailability in animals and has a low plasma protein binding rate, making it advantageous in terms of pharmacokinetics [39]. Both enrofloxacin and ciprofloxacin are second-generation fluoroquinolones antibiotics with similar structures and antimicrobial activities. Enrofloxacin is reportedly converted into ciprofloxacin in certain animals (e.g., pigs and cattle) through metabolism [40]. Moreover, enrofloxacin use in poultry production increases the fluoroquinolone resistance of *C. jejuni*, leading to unsuccessful treatment of infected humans through food [41,42]. In addition, as a result of administering 50 ppm of enrofloxacin to chicken, fluoroquinolone resistance rapidly increased and *gyrA* mutation was confirmed in multiple individuals [43]. According to the domestic nationwide antibiotic usage and resistance monitoring data, among quinolone drugs, ciprofloxacin was not sold between 2010 and 2022, but enrofloxacin was the most consumed, i.e., 27,138–58,003 kg per year. However, due to the ban on enrofloxacin use in poultry from 31 October 2021, the sales decreased to approximately 27 tons in 2022, compared to 58 tons in 2021 [44]. Due to the same ban, the resistance correlation to the fluoroquinolone ciprofloxacin should be continuously analyzed and the resistance correlation between poultry and patient isolates should also be monitored.

Tetracycline has been a widely used antibiotic globally due to its low cost and high convenience. The resistance rate, which was expected to decrease due to the ban on tetracycline use for growth promotion in the EU in 2006, increased from less than 30% in 2001 to 47% in 2013 in *Salmonella* Typhimurium isolated from pigs, potentially due to the increased therapeutic use [45]. However, our results demonstrated 32.3% of tetracycline resistance in human patients (Table 2). According to another domestic report, the tetracycline resistance rate between 2009 and 2013 was 74.6% for human isolates and 81.0% for animal isolates [35], and the tetracycline resistance rate among human isolates in Gyeonggi-do, Korea, in 2010 was 69% [46]. Additionally, according to a domestic report on poultry, the tetracycline resistance rate of poultry isolates in 2013 was 52% in chickens and 97% in ducks [38]. Poultry isolates from 2016 to 2017 showed a resistance rate of 27.8% in chickens and a resistance rate of 57.8% in ducks [21], suggesting that tetracycline resistance in humans and poultry is decreasing in Korea. This could be potentially due to the lack of tetracycline sales for livestock use between 2011 and 2020 and the 55% sales volume reduction of oxytetracycline, a tetracycline family member, during the same period [30]. As a result, consistent with the data from One Health antimicrobial-resistant organisms, the tetracycline resistance of *C. jejuni* isolated from poultry decreased rapidly from 76.7% in 2012 to 37.5% in 2021 [47].

The most prevalent clonal complexes (CCs) (CC21, CC22, and CC206) accounted for 61% of the isolates and the most prevalent STs were ST4253, ST22, and ST572, with 66, 50, and 28 isolates, respectively. The most dominant CC in all human isolates was CC21, comprising 12 STs, of which the most common was ST4253 (66/136, 48.5%), also reported from a human clinical *C. jejuni* population in Japan [48]. Furthermore, CCs such as CC22, CC45, CC48, CC354, and CC443 were frequently reported from poultry meat isolates in Korea [20,49]. This study also identified 50, 22, 11, 17, and 4 isolates of the aforementioned complexes, respectively (Table 3). In Korea, ST21 of CC21 appeared to be dominant in each region in the human patient-derived samples [49]. Our study demonstrated the dominance of ST4253 of CC21 in patient-derived samples.

Between 2012–2017, as a result of *C. jejuni* genotype analysis for domestic patients with diarrhea in the acute diarrhea laboratory surveillance project, the main CCs were CC21 (89/194, 45.9%), CC443 (17/194, 8.8%), and CC22 (8/194, 4.1%). CC206 was analyzed in one sample out of 194 (1/194, 0.5%) [50]. This study analyzed CC21 (136/371, 37.7%), CC22 (50/371, 13.8%), and CC206 (34/371, 9.4%) in order (Table 4). Therefore, CC21 prevalence was still predominant in patients with diarrhea for 10 years. In particular, the increase in CC22 and CC206 indicates changes in the domestic epidemic. CC22 has been reported in domestic chicken [21]. However, CC206 is a clone found only in human isolates that have not been previously reported in poultry. Therefore, the gradually emerging CC206 clone in Korea would require clinical monitoring in the future. Previous studies have also reported that CC21 is the most commonly isolated *C. jejuni* CC in China, including human and chicken isolates [11,51]. Moreover, CC21 has also been isolated and reported in ducks in Korea [20], thereby providing evidence that poultry is a *C. jejuni* reservoir and a main transmission route for human infection. Therefore, *C. jejuni* MLST profiles in Asian patients with diarrhea revealed that CC21 is the most commonly isolated in China [11,51], Japan [48], and South Korea. Concerning the *C. coli* strains, our MLST analysis revealed that 90% (9/10) belonged to CC828, while 10% (1/10) represented a novel and previously unreported CC in the MLST database (Table 3). The CC828 complex, comprising eight STs (ST830, ST854, ST860, ST872, ST9201, ST9867, ST12600, and ST12613) yielded the highest diversity. The high prevalence of the CC828 strains in this study was consistent with its worldwide distribution [52].

In Korea, several studies have reported *Campylobacter* spp. prevalence in poultry [20,21,23,49]. However, clinical *Campylobacter* characterization remains limited. In this study, we described the characteristics of antimicrobial susceptibility profiles and molecular typing of clinical *Campylobacter* isolates from patients with acute diarrhea in South Korea. Furthermore, we underpinned the high genetic diversity of *Campylobacter* isolates circulating in Korea. Systematic *Campylobacter* surveillance and molecular characterization could provide important information to prevent *Campylobacter* infections in patients with diarrhea.

## 5. Conclusions

Our study highlights the antibiotic resistance patterns and genetic diversity of 371 clinical isolates from patients with acute diarrhea treated at more than 100 medical institutions in South Korea. The investigated *Campylobacter* clinical isolates exhibited drug resistance mainly to fluoroquinolone and tetracycline, and they were highly susceptible to macrolides. In particular, fluoroquinolones-resistant *Campylobacter* strains are emerging worldwide, representing a significant problem in clinical treatment. Even after the ban on ciprofloxacin use in 2010 in South Korea, no rapid decline could be observed and high-level ciprofloxacin resistance was still identified. However, tetracycline resistance displays a decreasing trend. Macrolide and gentamicin need to be appropriately used to prevent increasing resistance against them in *Campylobacter* spp. The results did not meet our expectations that the ciprofloxacin resistance of *Campylobacter* isolated from patients and poultry would decrease after the ban on fluoroquinolone as a growth promoter in poultry in Korea. In addition, as enrofloxacin was banned by the Korean Government since 31 October 2021, we plan to conduct additional research to determine whether this ban has an impact on changes in fluoroquinolone resistance rates through patient–poultry genetic correlation analysis from a One Health perspective.

Moreover, we revealed the high genetic diversity of *Campylobacter* isolates circulating in South Korea. Our MLST analysis demonstrated that CC21 (ST4253), CC22 (ST22), and CC206 (ST572) were high-frequency clones in *C. jejuni*, CC828 was a dominant clone in *C. coli*, and identified eight novel STs. Therefore, it is important to continuously monitor the spread of *Campylobacter* resistance through antimicrobial susceptibility and molecular analysis of patient isolates, which have been lacking in studies. In conclusion, this study contributes to the establishment of basic data necessary to identify the correlation between clinical isolates and to prevent *Campylobacter* spread in the potential future event of a large-scale patient outbreak.

## Figures and Tables

**Table 1:** Results of PCR and culture tests for bacteria causing acute diarrhea requested from six regions from April to September 2022.

*Campylobacter* spp.	Acute Diarrhea Causative Bacteria PCR Test			Culture Test	
	No. of Requests	Positive Cases	Positive Rate (%)	Positive Cases	Isolation Rate (%) ^1^
Seoul	3322	132	4.0%	35	26.5
Gyeonggi	5877	555	9.4%	88	15.9
Chungcheong	4539	254	5.6%	39	10.9
Jeolla	8654	691	8.0%	75	15.0
Gyeongsang	10,032	749	7.5%	104	13.9
Gangwon/Jeju	1087	111	10.2%	30	27.0
Total	33,511	2492	7.4%	371	14.9%

^1^ Isolation rate (%) = Culture-positive cases/PCR-positive cases.

**Table 2:** Distribution of the MICs for *C. jejuni* (n = 361) and *C. coli* (n = 10) isolates from human patients.

Antimicrobial Agents ^1^		Species	No. of Isolates with MIC (µg/mL)											MIC_50_/MIC_90_^2^ (µg/mL)	Resistance Breakpoint ^3^ (µg/mL)	Resistance Rate (%)	
			≤0.06	0.125	0.25	0.5	1	2	4	8	16	32	64				
Macrolides	AZI	*C. jejuni*	359		1				1					≤0.06/0.125	≥0.5	0.3	0.53
		*C. coli*	9										1	≤0.06/0.125	≥1	10	
	ERY	*C. jejuni*	58	179	101	21	1		1					0.25/0.5	≥8	0	0.53
		*C. coli*		2		4	3						1	1/2	≥16	10	
Fluoroquinolones	CIP	*C. jejuni*	53	6		4	2	39	114	123	18	2		8/16	≥1	82.6	83.3
		*C. coli*							2	8				16/16	≥1	100	
Quinolones	NAL	*C. jejuni*							58	10	62	120	111	64/>64	≥32	64.1	64.9
		*C. coli*									1	2	7	>64/>64	≥32	90	
Lincosamides	CLI	*C. jejuni*	303	47	9			1			1			≤0.06/0.25	≥1	0.6	0.8
		*C. coli*	1	5	2	1			1					0.25/1	≥2	10	
Phenicols	FFN	*C. jejuni*	2	7	117	203	23	8	1					1/1	≥8	0	0
		*C. coli*				5	4	1						1/2	≥8	0	
Tetracyclines	TET	*C. jejuni*	166	71	5	5	1	4	4	22	21	30	32	0.25/64	≥2	27.7	32.3
		*C. coli*			2	1					1	2	4	64/>64	≥4	70	
Aminoglycosides	GEN	*C. jejuni*		201	154	5	1							0.25/0.5	≥4	0	0.53
		*C. coli*			6	2						2		0.5/64	≥4	20	

^1^ AZI, azithromycin; ERY, erythromycin; CIP, ciprofloxacin; NAL, nalidixic acid; CLI, clindamycin; FFN, florfenicol; TET, tetracycline; GEN, gentamicin. ^2^ MIC_50_ and MIC_90_ indicate the concentration (μg/mL) at which 50% and 90% of isolates tested were susceptible to the antimicrobial, respectively. ^3^ Epidemiological cutoff values set by the U.S National Antimicrobial Resistance Monitoring System (NARMS). According to NARMS guidelines, the number of resistant strains for each antimicrobial agent is indicated in gray shading.

**Table 3:** Antimicrobial resistance parameters of *C. jejuni* (n = 361) and *C. coli* (n = 10) isolates.

*Campylobacter* spp.	MDR ^1^ Phenotype	Antimicrobial Resistance Profiles ^2^	No. of Isolates	Rate (%)
*C. jejuni* (n = 361)		TET	100	27.7
		CLI	2	0.55
		AZI	1	0.27
		CIP, TET	111	30.7
		CIP, NAL	228	63.2
		AZI, CIP, NAL	1	0.27
		CIP, NAL, TET	82	22.7
		CIP, NAL, CLI	1	0.27
	MDR	CIP, NAL, CLI, TET	1	0.27
	MDR	AZI, CIP, NAL, TET	1	0.27
	MDR	AZI, ERY, CIP, NAL, CLI,	1	0.27
	MDR	AZI, ERY, CIP, NAL, CLI, TET	1	0.27
*C. coli* (n = 10)		TET	7	70
		GEN	2	20
		AZI, ERY	1	10
		CIP, TET	7	70
		CIP, NAL	10	100
		CIP, NAL, TET	7	70
	MDR	CIP, NAL, TET, GEN	2	20
	MDR	AZI, ERY, CIP, NAL, CLI	1	10

^1^ Multidrug-resistant. ^2^ AZY, azithromycin; ERY, erythromycin; CIP, ciprofloxacin; NAL, nalidixic acid; CLI, clindamycin; FFN, florfenicol; TET, tetracycline; GEN, gentamicin.

**Table 4:** *Campylobacter jejuni* (n = 361) and *Campylobacter coli* (n = 10) isolate sequence types.

*Campylobacter* spp.	Clonal Complex (CC)	ST	No. of Isolates		Rate (%)
*C. jejuni* (n = 361)	CC21 (n = 136)	19	4		37.7%
		21	15		
		50	26		
		298	8		
		451	4		
		760	1		
		806	4		
		982	2		
		3128	1		
		3293	1		
		4253	66		
		6500	4		
	CC22 (n = 50)	22	50		13.8%
	CC206 (n = 34)	122	2		9.4%
		227	1		
		572	28		
		3335	3		
	CC45 (n = 22)	45	8		6.0%
		137	2		
		538	1		
		583	10		
		3456	1		
	CC658 (n = 19)	523	2		5.3%
		1044	17		
	CC354 (n = 17)	12630	17		4.7%
	CC48 (n = 11)	48	9		3.0%
		918	2		
	CC61 (n = 11)	61	9		3.0%
		81	1		
		6129	1		
	CC42 (n = 9)	42	8		2.5%
		604	1		
	CC464 (n = 8)	464	7		2.2%
		8627	1		
	CC353 (n = 7)	5	3		1.9%
		1898	4		
	CC443 (n = 4)	51	3		1.1%
		5671	1		
	CC460 (n = 4)	460	4		1.1%
	CC508 (n = 4)	508	4		1.1%
	CC607 (n = 4)	904	2		1.1%
		3611	1		
		12635	1	assign	
	CC283 (n = 3)	4063	3		0.8%
	CC574 (n = 3)	305	2		0.8%
		12630	1		
	CC362 (n = 3)	12596	3	assign	0.8%
	NT ^1^ (n = 2)	12636	2	assign	0.5%
	CC49 (n = 1)	467	1		0.2%
	CC1034 (n = 1)	12640	1	assign	0.2%
	NT (n = 1)	12268	1		0.2%
	NT (n = 1)	12597	1	assign	0.2%
	NT (n = 1)	12656	1	assign	0.2%
	NT (n = 1)	2274	1		0.2%
	NT (n = 1)	4240	1		0.2%
	NT (n = 1)	5046	1		0.2%
	NT (n = 2)	5229	2		0.5%
*C. coli* (n = 10)	CC828 (n = 9)	830	1		90%
		854	1		
		860	2		
		872	1		
		9201	1		
		9867	1		
		12600	1	assign	
		12613	1	assign	
	NT (n = 1)	10873	1		10%

^1^ NT, non-typable CC.

## Data Availability

All data generated or analyzed during this study are included in this published article.

## Supplementary Materials

The following supporting information can be downloaded at: https://www.mdpi.com/article/10.3390/microorganisms12010094/s1. Figure S1: Prevalence of *Campylobacter* isolates investigated in this study.
