# Supplementary material for: Antimicrobial Susceptibility Patterns and Genetic Diversity of Campylobacter spp. Isolates from Patients with Diarrhea in South Korea
Source: Microorganisms. 2024 Jan 2;12(1):94. doi: 10.3390/microorganisms12010094 (PMC10819060; doi:10.3390/microorganisms12010094)
Supplement: Supplementary file 1 [file microorganisms-12-00094-s001.zip › microorganisms-2756200-supplementary.pptx]

## Slide 1
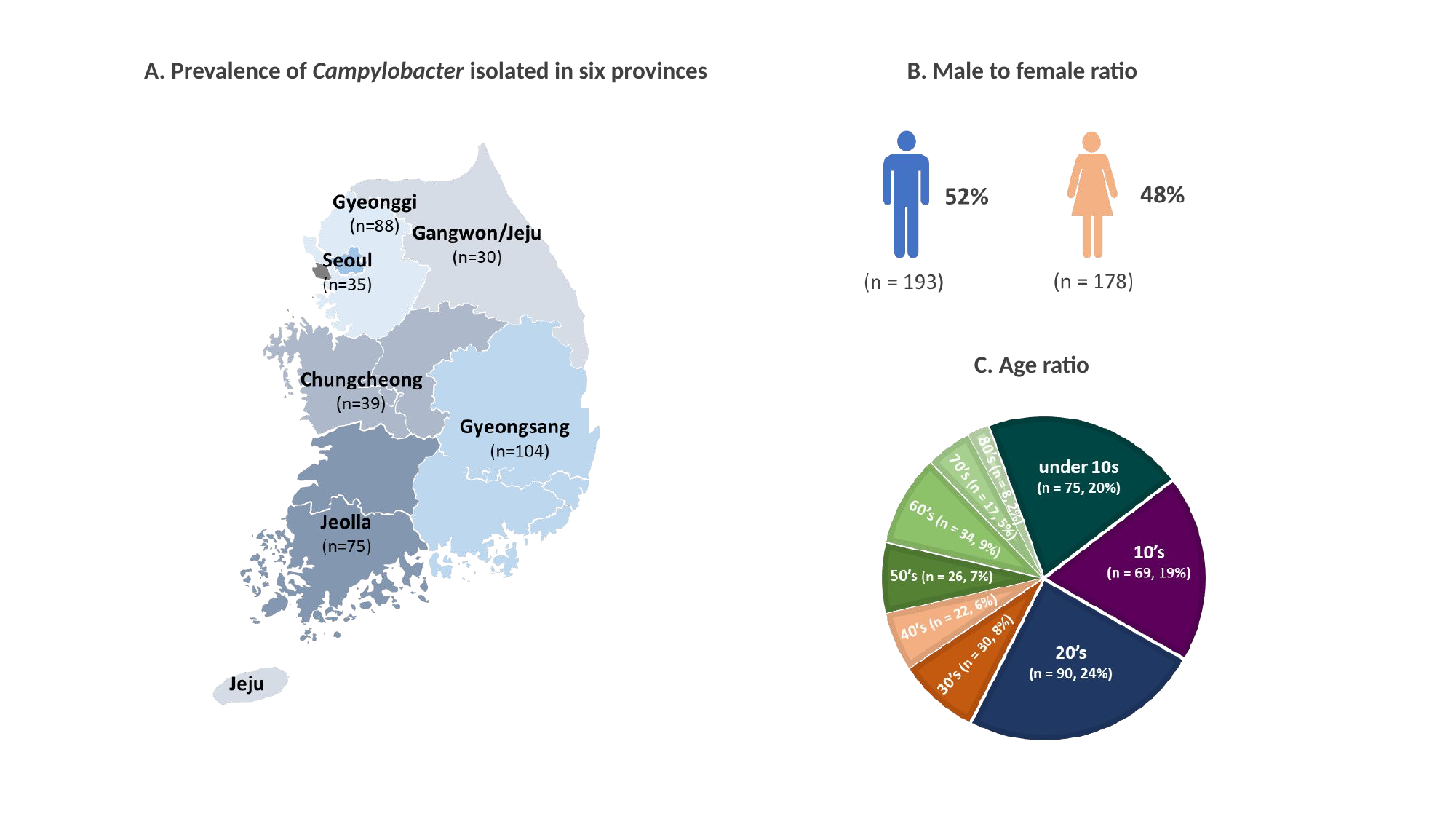

A. Prevalence of Campylobacter isolated in six provinces
B. Male to female ratio
C. Age ratio
